# The Effects of Chronic Psychostimulant Administration on Bone Health: A Review

**DOI:** 10.3390/biomedicines12081914

**Published:** 2024-08-21

**Authors:** Jessica Nowak, Jacob Aronin, Faraaz Beg, Natasha O’Malley, Michael Ferrick, Teresa Quattrin, Sonja Pavlesen, Michael Hadjiargyrou, David E. Komatsu, Panayotis K. Thanos

**Affiliations:** 1 Behavioral Neuropharmacology and Neuroimaging Laboratory on Addictions (BNNLA), Clinical Research Institute on Addictions, Department of Pharmacology and Toxicology, Jacobs School of Medicine and Biomedical Sciences, University at Buffalo, Buffalo, NY 14203, USA; 2 Department of Orthopaedics, University of Rochester Medical Center, Rochester, NY 14642, USA; 3 Department of Orthopaedics, Jacobs School of Medicine, University at Buffalo, Buffalo, NY 14203, USA; 4 UBMD Pediatrics, JR Oishei Children’s Hospital, University at Buffalo, Buffalo, NY 14203, USA; 5 Clinical Research Center, UBMD Orthopaedics & Sports Medicine, 111 N Maplemere Rd., Suite 100, Buffalo, NY 14221, USA; 6 Department of Biological and Chemical Sciences, New York Institute of Technology, Westbury, NY 11568, USA; mhadji@nyit.edu; 7 Department of Orthopaedics and Rehabilitation, Stony Brook University, Stony Brook, NY 11794, USA

**Keywords:** methylphenidate, amphetamine, ADHD, osteoblast, osteoclast, dopamine

## Abstract

(1) Background: Methylphenidate (MP) and amphetamine (AMP) are psychostimulants that are widely prescribed to treat Attention Deficit Hyperactivity Disorder (ADHD) and narcolepsy. In recent years, 6.1 million children received an ADHD diagnosis, and nearly 2/3 of these children were prescribed psychostimulants for treatment. The purpose of this review is to summarize the current literature on psychostimulant use and the resulting effects on bone homeostasis, biomechanical properties, and functional integrity. (2) Methods: Literature searches were conducted from Medline/PubMed electronic databases utilizing the search terms “methylphenidate” OR “amphetamine” OR “methylphenidate” AND “bone health” AND “bone remodeling” AND “osteoclast” AND “osteoblast” AND “dopamine” from 01/1985 to 04/2023. (3) Results: Of the 550 publications found, 44 met the inclusion criteria. Data from identified studies demonstrate that the use of MP and AMP results in decreases in specific bone properties and biomechanical integrity via downstream effects on osteoblasts and osteoclast-related genes. (4) Conclusions: The chronic use of psychostimulants negatively affects bone integrity and strength as a result of increased osteoclast activity. These data support the need to take this into consideration when planning the treatment type and duration for bone fractures.

## 1. Introduction

Psychostimulants such as methylphenidate (MP) and amphetamine (AMP) are widely prescribed to treat Attention Deficit Hyperactivity Disorder (ADHD) and narcolepsy, with rising rates of nonmedical use also reported [1,2]. The Diagnostic and Statistical Manual of Mental Health Disorders, Fifth Edition (DSM-V) identifies three types of ADHD that are characterized by presented symptoms: predominantly inattentive, predominantly hyperactive/impulsive, or a combined presentation [3]. Along with the presenting characteristics, an increase in inappropriate and disruptive behavior relative to age is also taken into account, with prevalence higher in males [4,5]. ADHD is associated with Reward Deficiency Syndrome (RDS), a dysfunction within the brain’s reward system that is modulated by neurotransmitters, including dopamine (DA) [6,7]. In 2016, 6.1 million children in the United States aged 2–17 had ever received an ADHD diagnosis [8]. 

In recent years, it has been noted that 62.0% of children diagnosed with ADHD were taking medication [8]. Side effects of these medications include a reduction in appetite, insomnia, and rare psychiatric or cardiac issues [9]. The long-term use of stimulants has also been associated with decreased height gain and weight suppression in some children [10]. Daily treatment with MP for consecutive years leads to lower-than-predicted height and weight [11] in a dose-dependent manner [12]. It was also reported that intermittent breaks from psychostimulant use, such as “summer breaks”, allow recovery of height and weight gain, such that no differences are observed in comparison to individuals not using psychostimulants [13]. As such, stimulants have gained popularity due to their positive effects on mood, prolonged ability to stay awake, and increased concentration [14]. The range of nonmedical use of prescription stimulants (NUPSs) is reported to range from 8 to 17% of students [15,16], and as such, it is a growing societal concern. 

Although the majority of studies focus on the effects of psychostimulants on the brain and behavior, it behooves us to also examine the impact these drugs have on other organs. Bone health with fracture risk and healing relating to ADHD has started to be better understood in recent years as well. While psychostimulants appear to impact bone healing following traumatic fracture, a Mendelian randomized study identified ADHD as a risk factor for fracture of the ribs, foot, ankle, lower leg, wrist, and hand [17]. Between increased prescribed psychostimulant use and NUPS, it is pertinent to understand the relationship between psychostimulant use and skeletal health. Thus, in this comprehensive narrative review, we focus on the effects of psychostimulants on bone health, including both animal and human studies. This includes information regarding AMP and MP mechanisms of action, the behavioral effects of psychostimulants on rodents, and effects on bone health, including DA regulation in osteogenic cells, pathways, gene expression, bone structure, and reported effects in humans.

## 2. Literature Criteria for Inclusion

**Figure 1:**
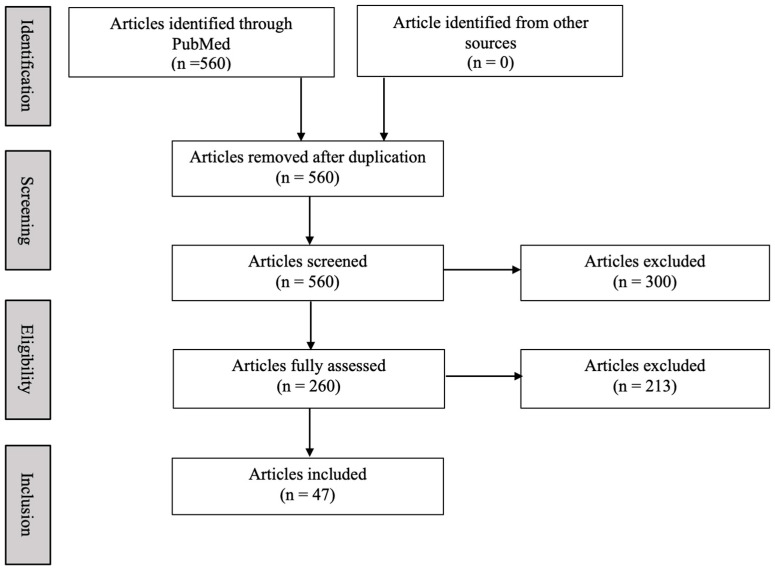
PRISMA flow chart. Number of articles identified, screened and included in this review.

We searched PubMed and Web of Science electronic databases utilizing the search terms “methylphenidate” OR “amphetamine” OR “dopamine” AND “bone health” AND “bone remodeling” AND “osteoclast” AND “osteoblast”. Articles were included if they met the following inclusion criteria (Table 1): (1) English language; (2) publication date from January 1985 to April 2023; (3) psychostimulants MP and AMP only; (4) peer-reviewed articles including pediatric and/or adult population studies, rat and/or mice studies, review papers and meta-analyses; (4) outcomes related to bone health including biomechanical integrity, growth suppression, bone mineral content, and bone mineral density; and (5) outcomes related to mechanisms including osteoblasts, osteoclasts, and dopamine receptors. Case studies and editorials were excluded. Three reviewers independently screened the titles and abstracts of all articles identified from the electronic database searches. Additionally, if it was unclear from the title and/or abstract whether the article should be included, the reviewers independently assessed the full text of the articles for inclusion and exclusion criteria (Table 1). Any discrepancies were resolved by consensus. The exact search syntax was provided, and a PRISMA flow chart was constructed (Figure 1). Furthermore, we created tables to establish requirements for data extraction from the included articles. This search and application of inclusion/exclusion criteria yielded a total of 47 articles.

## 3. Mechanism of Action

### 3.1. AMP

**Figure 2:**
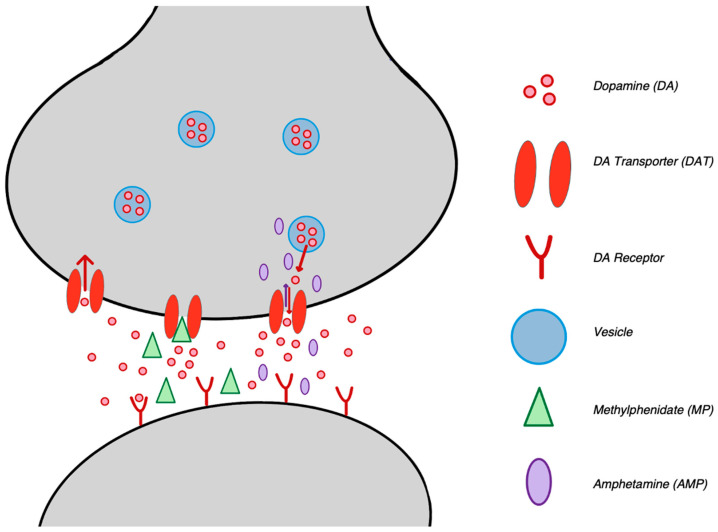
Mechanisms of AMP and MP on the DAT. Normal-functioning DAT reuptakes extracellular DA from the cleft; MP acts as a transporter blockade and blocks the reuptake of DA, resulting in an increase in DA levels at the synapse; and AMP acts as a transporter reversal, resulting in an efflux of DA back into the synaptic cleft.

As monoamines, AMP and MP act as substrates to monoamine transporters for the dopamine transporter (DAT), norepinephrine transporter (NET), and serotonin transporter (SERT) [18]. The main function of the DAT is to remove extracellular DA from the synaptic cleft (Figure 2) [19,20,21,22,23]. AMP has effects on monoaminergic systems and secretory vesicles, DA synthesis, and the activity of degradative enzymes [20]. AMP acts as a transporter reversal, passing through DAT as a substrate, resulting in an efflux of DA back into the synaptic cleft (Figure 2) as well as other monoamine transporters [18,21,23]. The secondary mechanism of AMP related to DA is the vesicular monoamine transporter (VMAT). While the function of DAT is to remove DA from the synaptic cleft, the function of VMAT is to move the recently cleared DA from the cleft to the presynaptic vesicle and store the DA in secretory vesicles until it is ready for release [20,21,24]. Lastly, AMP has optically active enantiomers, including *d-AMP* and *l-AMP*, which are found in a 3:1 ratio of d-AMP:l-AMP in the medication Adderall ^®^, making it a racemic mixture [22,25].

### 3.2. MP

Similar to AMP, MP results in an increase in DA concentration in the neuronal synapses. Despite having the same effect, MP functions via a more direct mechanism by acting as a transporter blockade (Figure 2) [26,27]. Specifically, MP binds to DAT and inhibits presynaptic reuptake, allowing for an increased DA concentration within the synaptic cleft [28,29,30]. Like AMP, MP is not selective for DAT and also acts as a transporter blockade for other monoamine transports like NET and SERT [26,29]. Although MP does not display specificity in transporter selection, there is a difference in the affinity between MP and the transporters; this is greatest for DAT, followed by NET, and lowest for SERT [27,31,32]. Specifically, it was reported that the affinity of MP to DAT was nearly 10-fold higher than NET [26]. Notably, the mechanism of MP and these transporters are similar to cocaine, which also acts a monoamine transporter blocker [31,33,34].

## 4. Behavioral Effects of Psychostimulants in Rodent Models 

Understanding the mechanisms and outcomes of AMP and MP relative to DA becomes useful in understanding the behavioral effects in rodent models. Yan et al. (2011) reported that the ADHD characteristics in a mouse model lacking the neurkinin-1 receptor disappeared following treatment with either AMP or MP [35]. Similar behavioral results were observed with psychostimulants in DA D1, D2, or DAT knockout mice [36,37,38]. A comparison of healthy adults and a non-ADHD mouse model showed that both humans and mice had increased locomotive response to AMP in a dose-dependent manner [39]. In addition, MP administration had a dose-dependent and strain-dependent effect on decreasing impulsivity in several rat models of ADHD [40,41,42]. 

Specifically, the ADHD rodent model utilized is the spontaneously hypertensive rat (SHR), as these animals display hyperactivity, impulsivity, and inattention compared to Wister–Kyoto (WKY) rats. The WKY strain is the one that SHRs were derived from, and thus, this group functioned as non-ADHD controls [43,44,45]. SHRs display neurophysiological differences similar to those seen in humans with ADHD, including decreased prefrontal cortex and hippocampus volumes [44,46]. Studies of SHRs treated with AMP or MP are not conclusive regarding behavior as some studies showed a decrease in ADHD behaviors [44] while others have shown an increase in hyperactivity [46]. Other findings, such as improved short-term memory, hyperactivity, and attention in SHRs following AMP administration, support the validity of this model [43,47,48,49,50].

A second rodent model of ADHD is DAT KO mice [46]. These mice display attenuated hyperactivity following the administration of AMP or MP [44,45] and represent a more extreme model of ADHD, which was used to generate DAT knockdown (KD) mice. DAT KD mice expressed 10% of DAT [50] compared to WT mice and exhibited the same hyperactivity and impaired response inhibition as DAT KO mice without reduced growth and premature death. DAT KD mice also displayed reduced hyperactivity following the administration of DR1/2 and DR2 agonists [46].

LPHN-3 is a member of a sub-family of G-protein-coupled receptors involved in monoamine signaling [45]. Rats lacking LPHN-3 display hyperactivity along with attention and inhibitory control deficits. Additionally, these rats continued to display hyperactivity following AMP administration; however, it was reduced compared to the baseline. Conversely, wildtype rats displayed increased hyperactivity following AMP administration [51]. Collectively, LPHN3 KO rats represent a potential new model for hyperactive/impulsive ADHD [45]. 

Finding the appropriate rodent models for understanding the behavioral side effects of psychostimulant medications could also potentially translate to a better understanding of skeletal effects. Understanding the mechanism behind ADHD-like characteristics and DA levels within models can aid in further understanding DA levels in relation to negative skeletal effects. 

## 5. Reported Effects of Psychostimulants on Bone Properties

Although the efficacy of psychostimulant treatment for ADHD has been well-documented, the adverse skeletal effects of these treatments have not been thoroughly explored. Despite being the recommended treatment for ADHD, the use of psychostimulants has been associated with growth suppression and reduction in bone mass through the dysregulation of the bone remodeling cycle [52,53,54,55,56]. Treatment with MP resulted in decreased bone mineral content (BMC) and bone mineral density (BMD) in rats, but these effects were mitigated following the discontinuation of MP [52,57]. Similarly, children taking psychostimulants were shown to have decreased BMD and BMC compared to nonusers [58]. Additionally, growth suppression has been observed in school-age children taking psychostimulant medication [59,60]. 

The key cells responsible for bone growth and homeostasis are osteoblasts, osteocytes, and osteoclasts. Osteoblasts are the cells responsible for bone growth and are derived from mesenchymal stem cells. Following differentiation into mature osteoblasts, these cells secrete osteoid, which is the mix of extracellular matrix (ECM) that becomes bone. The primary ECM protein is type 1 collagen, which represents 95% of the bony matrix. Collagen serves as a scaffold for the deposition of hydroxyapatite, while the inorganic component of bone is predominantly composed of calcium and phosphate. After reaching maturity, osteoblasts can be removed via apoptosis, revert to a quiescent state as bone-lining cells, or further differentiate into osteocytes. 

Osteocytes are embedded in the bone matrix and represent numerous bone cells, accounting for over 95% of adult bone cells. They reside in cavities known as lacunae and are interconnected with other osteocytes via channels known as canaliculi. The main function of osteocytes is to regulate skeletal homeostasis. They do so by directly sensing mechanical signals, as well as responding to paracrine and endocrine signals and then releasing factors that activate or inhibit osteoblasts and osteoclasts. 

Osteoclasts are the last main type of bone cell. In contrast to osteoblasts and osteocytes, osteoclasts are derived from hematopoietic stem cells and during differentiation they fuse to become multinucleated mature osteoclasts. Mature osteoclasts are polarized, with a smooth membrane on their apical side and a ruffled border on their basal side. This ruffled border forms a strong seal with the bone below and the osteoclasts secret acidic components and proteases into this space to resorb bone and ECM. To maintain homeostasis, osteocytes precisely regulate osteoblastic bone formation and osteoclastic bone resorption through a process known as coupled remodeling. This allows for the replacement of damaged bone. Similarly, osteocytes can shift the balance of formation and resorption in response to anabolic and catabolic stimuli, respectively. Understanding the functions and homeostatic relationship of the cells of bone are important for research surrounding the regulation of bone.

### 5.1. Dopaminergic Regulation of Osteogenic Cells 

The direct effects of AMP and MP use are increased DA with a concomitant increase in DA receptor activation [32]. Table 2 summarizes the physiological effects of cell lines with excess DA exposure. DA receptors are G protein-coupled receptors (GPCRs) that aid in the regulation of secondary messenger cyclic adenosine monophosphate (cAMP) levels [61,62]. There are five known DA receptors (D1R-D5R) that are categorized by regulatory and structural characteristics. The D1R-like family is composed of D1R and D5R and upregulates cAMP; it has a long C-terminal domain, whereas the D2R-like family is composed of D2R, D3R, and D4R, downregulates cAMP and has a large intracellular loop between transmembrane proteins 5 and 6 [61,62,63]. The expression of all five DA receptors was found on both osteoblasts and osteoclasts, yet the DA regulation of these cells was different [64,65]. The activation and inhibition of D1R have regulatory effects on osteoblasts [61,66], while only D2R is associated with osteoclasts [65,67]. Furthermore, studies relating decreased osteoblastogenesis and increased osteoclastogenesis to the physiological conditions of Parkinson’s disease reinforce the understanding that DA plays a role in skeletal health [68]. 

### 5.2. Pathways

**Figure 3:**
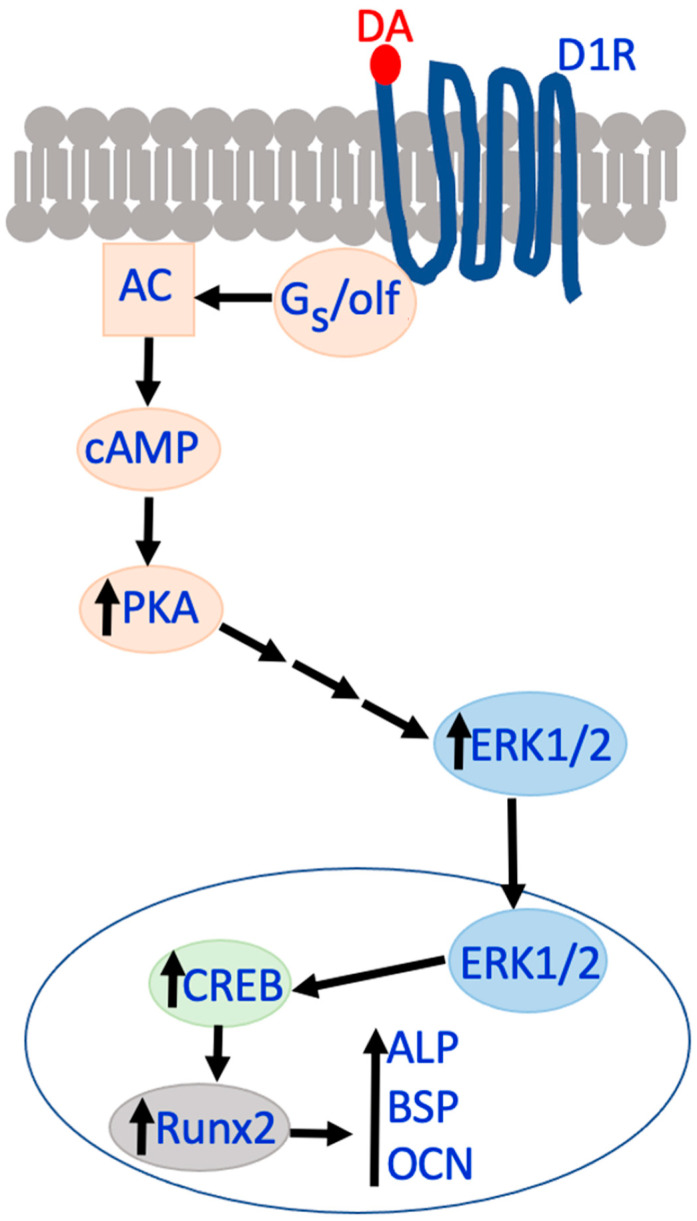
D1R-Runx2 activation pathway. In vitro pathway associated with D1R activation and downstream increases in cAMP, PKA, ERK1/2, Runx2, and osteoblast genes ALP, BSP, and OCN.

D1R activation and expression are associated with the osteogenic differentiation of multipotent mesenchymal stem cells (MSCs) into osteoblasts, and one of the associated pathways is D1R-cAMP-PKA-ERK1/2-Runx2 (Figure 3) [61,62,71]. Briefly, D1R activates PKA via cAMP, which then phosphorylates ERK1/2 and ultimately induces the expression of the master osteoblast transcription factor, Runx2 [61]. Previously, it was shown how chronic treatment with MP increases phosphorylation of ERK1/2, but acute exposure does not cause changes in ERK1/2 phosphorylation in rat brains [63]. This may suggest a direction for future human studies, such as identifying the duration of exposure that will induce this effect, as it was shown to be transient and reversible. This is most pertinent to school-age children, as a common phenomenon is a ‘drug holiday’ during the summer; that is, stopping the use of medication during the academic summer break. Additionally, there is evidence of D1R damage from chronic, repeated MP treatment in mice [72].

**Figure 4:**
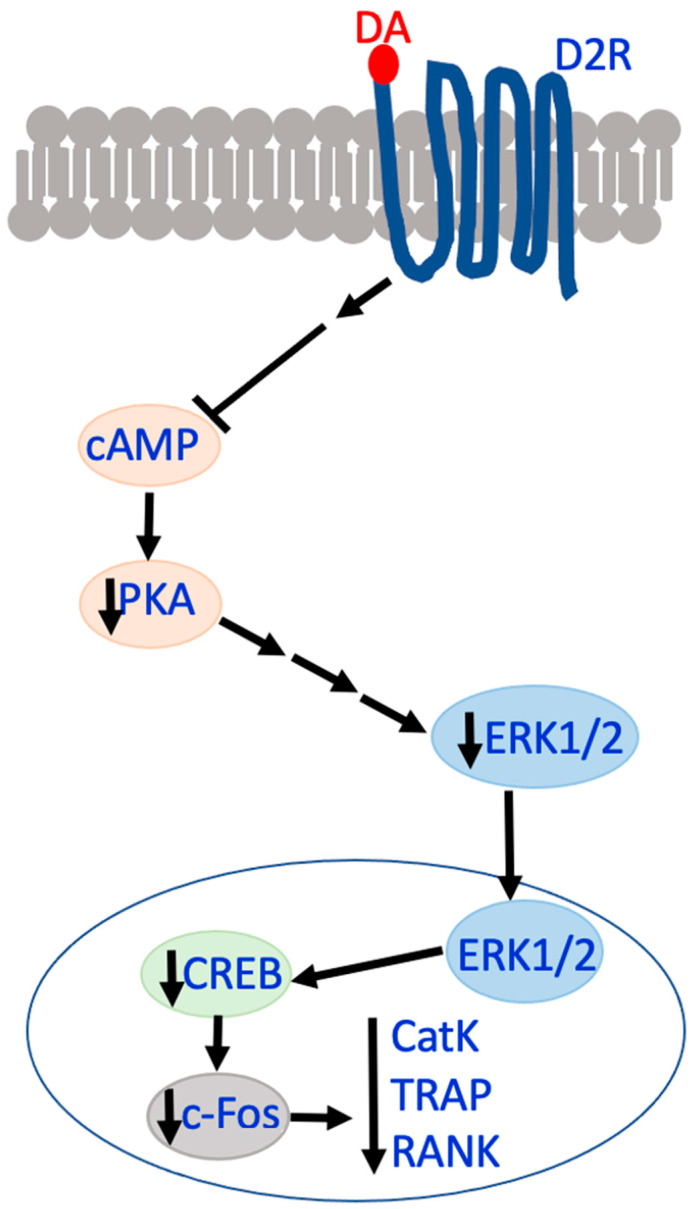
D2R-CREB activation pathway. In vitro pathway associated with D2R activation and a downstream decrease in cAMP, PKA, CREB, and osteoclast genes, such as c-Fos.

The activation of D2R does not exhibit the same effects on ERK1/2 phosphorylation and osteoblast differentiation as D1R [61]. Instead, the activation of D2R with DA is associated with the inhibition of osteoclast differentiation [62,65]. Compared to the D1R activation pathway, the associated D2R pathway is D2R-cAMP-PKA-CREB (Figure 4) [65]. In contrast to the D1R pathway, the DA activation of D2R decreases cAMP, followed by the downregulation of PKA and CREB, which is similar to Runx2 and acts to regulate osteoclast-related genes. One study noted that stimulation of D2R had a positive effect on osteoclastogensis; however, it did not induce osteoclast activation and resorption [69]. Notably, using a D2R-like antagonist simulated increasing cAMP levels and resulted in the increased activation of the cAMP-PKA-CREB pathway, which caused a reversal in the DA-induced inhibition of osteoclastogenesis [62,65].

Other pathways that could be considered include the Wnt/beta-catenin pathway, as a recent study showed that decreased Wnt/beta-catenin signaling via decreased osteoblast signaling resulted in increased osteoclast differentiation [73]. Other pathways that could be further explored are those that influence nuclear factor-activated T cell 1 (NFATc1), a key transcription factor in osteoclastogenesis, including RANK/RANKL and Ca^2+^-related signaling [74]. Considering that osteoblasts and osteoclasts have separate lineages, including mesenchymal stem cells and hematopoietic/macrophage, respectively, it would be advantageous to further explore how DA affects the associated pathways of each lineage. For example, it was recently noted that hematopoietic stem and progenitor cells are regulated by the D2R-cAMP-PKA-Lck protein pathway [67]. Additionally, local concentrations of DA have been noted to modulate monocyte-derived macrophage activity [75,76]. Other recent studies have shown that dopamine does have a regulatory effect on the peripheral immune system, including macrophages [77,78]. However, since the regulation of both of these cells is relative to each other, it is also important to understand how downstream pathways affect the differentiation and activation of the other cell.

### 5.3. Gene Expression

The decreased bone health observed after exposure to psychostimulants is the result of transcription factors that are affected by the cascade of the DA-response system [79]. Table 3 summarizes the effects on osteogenic genes in response to DA, AMP, and MP exposure. CREB is important for the activation of osteoclastic genes involved in differentiation. These genes include c-Fos and Stat3, as they have a cAMP response element (CRE) in their promoter regions [65]. AMP was shown to increase c-fos expression in the striatum but not increase CREB expression. The DA-induced effect of CREB activation was seen only when CREB was in a complex with regulatory elements, which created a temporary increase in c-fos expression, as regulated by D1R and D5R [79].

Other osteoclast-related genes that are downregulated by the DA activation of the D2R-like family or D2R-like agonists include cathepsin K, NFATc1, and ZIF268 [65]. ZIF268 or ERG1 is a zinc finger transcription factor that functions as a gene regulator by activating immediate–early genes that act in rapid response to stimuli [80]. Like AMP, MP has been shown to increase the expression of both c-fos and ZIF268 in the striatum [72]. In terms of osteoblast-related genes, Runx2 is a transcription factor that binds to the promotor regions of various osteogenic genes required for osteoblast differentiation. Genes that increase their expression in response to Runx2 elevation include BSP, ALP, and OCN. OSX is another transcription factor associated with osteoblast differentiation, but its expression is not associated with Runx2 [61]. 

### 5.4. Bone Structure

Table 4 summarizes the effects of psychostimulants noted using animal lines. Studies conducted in our laboratories [57] demonstrated the effects of psychostimulants on bone; specifically, the treatment of male rats with MP resulted in impaired femoral biomechanical properties in a dose-dependent manner, and these effects were abolished following a recovery period [52,57]. Furthermore, the rats treated with a high dose of MP also experienced reductions in BMD and BMC in the femur and tibia, which were also abolished following a recovery period [57]. There was also a dose-dependent decrease in anterior–posterior diameter in the femur, which also resolved following a recovery period. Interestingly, these impairments were restricted to the appendicular skeleton, with no changes seen in the axial skeleton [57]. This study also showed that ALP levels were increased in a dose-dependent manner during recovery, suggesting that osteoblast activity increased during the recovery period. 

A follow-up study showed that high or low doses of MP in rats resulted in dose- and sex-dependent effects on bone and osteoclast function [81]. The high-dose male group exhibited decreased femoral biomechanical integrity; however, females exhibited no statistically significant changes in biomechanical integrity [81]. It was also shown that the high-dose male group had reduced trabecular BMD and increased osteoclast numbers, whereas females had no changes. We then evaluated male and female osteoclasts in vitro and found that MP directly promoted osteoclast differentiation and resorption in osteoclasts derived from male but not female rats. Taken together, these data suggest that MP directly promotes osteoclastogenesis in male but not female rats. 

Depression and anxiety are known comorbidities of ADHD, and a recent study using the combined treatment of these conditions potentiated the effects on bone [82]. Specifically, male rats chronically treated with MP and/or fluoxetine (FLX) revealed the decreased length of the appendicular skeleton along with the anterior–posterior and medial–lateral diameter of the bone with MP + FLX treatment, leading to the most significant impairment [82]. The biomechanical properties of their bones were also decreased with treatment, again, with MP + FLX showing the most impairment compared to only MP or FLX treatment [82]. However, these results are somewhat confounded by the strong anorexigenic effects seen in the MP + FLX group.

Based on these studies, we hypothesized that excess DA from chronic psychostimulant use resulted in an imbalance of the osteoclast and osteoblast function, leading to decreased biomechanical properties. Previously, it was reported that osteoblast activity decreased in the presence of excess DA [61]. Although in vitro studies of DA exposure on osteoclasts showed a decrease in osteoclast function [65], in vivo studies suggest that AMP, DA, and MP increase osteoclast function in a dose-dependent manner [62,72,79]. Several studies have suggested that increased levels of cAMP from increased D1R/D5R-phosphorylated PKA and CREB reverse the osteoclast inhibition exhibited in vitro [62,79].

### 5.5. Reported Effects in Humans

Treatment with psychostimulants appears to attenuate height in humans; however, the cessation of medication allows growth to recover [83]. Table 5 summarizes the noted effects of psychostimulant use within humans. Decreased BMC and BMD were also detected following as little as three months of MP and AMP use in children and adolescents [58]. This loss was observed more at sites of trabecular bone in the lumbar spine and femoral neck [58]. Psychostimulant use was also found to have a negative effect on the healing process following traumatic fractures in a pediatric population. This effect was most significant within those with three to five years of use, and no effect was seen with more than five years of use [84]. Considering that ADHD can be treated in conjunction with other conditions, a study examined male children treated with psychostimulants along with risperidone. The results found no statistically significant decrease in BMD in the lumbar spine or distal radius; however, boys who used MP before and throughout the duration of the study did have reduced height [85]. Another study concluded that only a minor difference in BMD in the skull and thoracic spine was seen in adults using psychostimulants [86]. The increased incidence of stress fractures with the chronic use of psychostimulant medication has also been noted [87]. 

## 6. Conclusions

The data reviewed herein highlight the effects of psychostimulants on bone. Previous research [28] concluded that psychostimulants such as AMP and MP affect bone, especially during the remodeling cycle. The effects that these psychostimulant drugs have on the DA levels in the body directly affect how osteoblasts and osteoclasts function during remodeling. The negative effects of psychostimulants, coupled with their potential misuse, increase the detrimental effects on the skeleton. Further, with the rise in ADHD diagnoses [3], there is the possibility of increased psychostimulant availability, leading to widespread negative skeletal effects. These effects suggest that further investigation of psychostimulants and the skeleton is needed, as well as an examination into whether children with a suggestive impairment of biochemical properties or bone quality should continue to receive them. ADHD and its treatment are fairly well-documented, but the direct effects of psychostimulants on overall bone health have not been thoroughly explored. A pressing future research question is how these medications may affect different age groups since these drugs are taken by children, adolescents, and adults. 

Further studies on the addictive aspects of psychostimulants and how to circumvent them, either through additional treatment or finding alternative drugs, may help to prevent their misuse and their detrimental effects on the mammalian skeleton. Additionally, further research around such comorbidities, including anxiety and depression, associated with ADHD and the treatments of these conditions may also help in finding the most attractive treatment options for each individual with limited adverse skeletal effects. For instance, managing these comorbidities was found to have increased healthcare costs and resource utilization [89] and made patients more likely to experience treatment changes [90] compared to those without additional psychiatric comorbidities. Finally, studies of osteoblast and osteoclast function in response to excess DA concentration, DA receptor activation as well as signaling pathways could also be beneficial to understanding these links. 

Limitations of this study include the selection of only English-published studies related to chronic psychostimulant use and its effects on the skeleton, as well as the fact that human studies are predominantly retrospective, with only a few longitudinal studies. Prospective randomized controlled trials are needed to establish a causal link and temporality between chronic psychostimulant (MP or AMP) use and impaired properties of bone.

## Figures and Tables

**Table 1:** Inclusion criteria for articles.

Criteria	Decision
Articles published in English	Inclusion
Published as a scientific peer-reviewed journal article	Inclusion
Keywords defined previously found in titles and/or abstracts	Inclusion
Primary research, meta-analysis and review papers	Inclusion
Papers published before 1985	Exclusion
Case studies and editorials	Exclusion

**Table 2:** Physiological effects of cell lines following exposure to increased dopamine (DA) concentrations and the dopamine receptor antagonist/agonist.

Reference	Subject	Study Design	Findings
Fernandez et al. [61]	Human and rat bone mesenchymal stem cells (BMSCs)	BMSC were subject to varying concentrations of DA during proliferation and differentiation and were then analyzed for ALP, q-PCR, Western blot, and chromatin immunoprecipitation	Osteogenesis of osteoblasts are reliant on D1R; hBMSC, subjected to an increased concentration of DA, resulted in an inhibitory effect of osteogenesis, similar to blocking the cAMP-PKA pathway
Wang et al. [62]	RAW 264.7 osteoclast cell line; C57BL/6J mice and bone marrow cells	RT-qPCR, Western blot, CREB luciferase reporter assay and cAMP immunoassay detection were carried out on cells after differentiation following exposure to DA and the D1R/D2R agonist/antagonist	DA suppressed osteoclast differentiation in vitro via D2R while the activation of the cAMP/PKA/CREB pathway reversed osteoclast inhibition
Lee et al. [64]	MC3T3-E1 pre-osteoblasts	Pre-osteoblasts were subjected to RT-PCR and Western blot analysis following DA exposure	Osteoblasts are under the control of the D1R receptor through in vitro investigation. DA is a positive modulator of OB gene expression; however, increased concentrations of DA causes toxicity
Hanami et al. [65]	Human CD14+/osteoclast	CD14+ cells were subject to osteoclast differentiation and testing, such as immunofluorescence staining, TRAP staining, pit formation assay, cAMP and osteoclast gene expression	D2R signaling inhibits osteoclast differentiation, cAMP formation, and c-fos expression
Schwendich et al. [69]	Peripheral blood mononuclear cells (PBMC), human osteoblast, and bone matrix	Cells were cultured with the D1R or D2R agonist and treated with DA followed by testing including TRAP staining, bone resorption assay	Bone matrix treatment with D2R agonist increased mineralization; PBMC cells treated with D1R agonist inhibited osteoclastogeneis, whereas the D2R agonist increased. DA receptor stimulation had no effect on OC activity
Moytl et al. [70]	MC3T3-E1 pre-osteoblast; bone marrow stromal cells	MC3T3-E1 cells were cultured and treated with DA and/or risperidone (RIS) followed by qRT-PCR and staining; bone marrow stromal cells were treated with RANKL and M-CFS and subjected to DA and or RIS	DA suppressed Mc3T3-E1 mineralization and gene expression; DA suppressed OC differentiation
Sun et al. [71]	PDLSC	Cells were treated with DA and subjected to alizarin red-staining, qRT-PCR, and Western blot	Osteogenic protein expression is most effective at optimally determined concentrations; the ERK1/2 pathway was promoted by DA

**Table 3:** Bone-related gene expression related to DA, AMP, and MP treatment.

Reference	Genes	Study Design	Summary Findings
Wang et al. [61]	DRD1 DRD2 OCN BSP RUNx2 ALP OSX	Human BMSD incubated with DA and subjected to RT-PCR, Western blot, CHiP analysis, and the D1R agonist SKF-38393	50 μM of DA promoted cell proliferation and the expression of BSP, ALP, Runx2, and OCN whereas 500 μM of DA significantly inhibited cell proliferation and associated genes; osteogenic markers were upregulated by SKF-38393; cAMP-PKA signaling suppression inhibited osteogenic differentiation; and SKF-38393 increased the activation of ERK1/2 and expression of Runx2, BSP, ALP, OCN, but not OSX
Wang, et al. [62]	CRB C-FOS STAT3 NFATC1 TRAP CTSK	RAW cells treated with DA were observed for OC differentiation, and genes followed via treatment with the D2R agonist or quinpirole	DA treatment inhibited the differentiation and expression of osteoclastic genes in a dose-dependent manner; similar effects were observed for quinpirole with reduced CREB phosphorylation; the blocking of D2R abolished the inhibitory effect; the decreased expression of cAMP, PKA and p-CREB due to dopamine was reversed with the activation of cAMP
Comim, et al. [63]	ERK1/2	Male Wistar rats were treated with MP for 28 days or 1 day and subjected to striatum dissection	Chronic treatment of MP increased the phosphorylation of ERK1, whereas acute exposure had no effect on the phosphorylation of ERK1/2
Konradi, et al. [79]	CaRE ATF	Male Sprague-Dawley rats were treated with AMP and subjected to further testing	fos protein expression increased after AMP exposure, but CREB did not; CREB expression only increased when bound to regulatory elements of c-fos; CREB expression is dependent on D1R/D5R
Brandon, et al. [72]	C-FOS ZIF268 DYNORPHIN	Male Sprague-Dawley rats were treated with MP and subjected to in situ hybridization histochemistry	MP exposure produced a dose-dependent increase in expression of c-fos and zif268, and chronic, repeated exposure resulted in D1R damage due to enhanced dynorphin expression
Hanami, et al. [65]	cAMP C-FOS NFATc1 CTSK	Human CD14+ were differentiated into OC precursor cells and treated with DA, and the D2R-like agonists, pramipexole and quinpirole	DA, pramipexole, and quinpirole produced the same effects of decreased osteoclast differentiation and expression of the osteoclast-related proteins CTSK, NAFTc1, cAMP, and C-FOS.

**Table 4:** Reported effects of psychostimulant use within animal lines relating to dopamine receptor (D1R, D2R) expression/activity, gene/pathway expression, and bone biomechanical properties.

Reference	Subject	Study Design	Findings
Komatsu, et al. [57]	Rat	SD rats were treated dose-dependently with MP and assessed for bone size, BMD, BMC, biomechanics, and serum biomarkers	The diameter of femoral bone decreased in a dose-dependent manner, along with BMD, BMC, biomechanical properties, and serum alkaline phosphatase levels that were abolished under the recovery protocol.
Comim, et al. [63]	Rat	Young male Wistar rats were subjected to MP and then decapitated	Chronic treatment of MP significantly increased ERK1/2 phosphorylation
Zhu, et al. [66]	Mice and osteoblast	BMSC and MC3T3-E1 cells were subjected to the D1R agonist and/or Dex then tested via staining and Western blot; C57BL/6J mice were divided into a control, vehicle, Dex + D1R agonist, Dex + D1R agonist + D1R inhibitor or Dex + D1R inhibitor group, and then tested through micro-CT and analyses	The overexpression of D1R reserves the inhibition of osteoblasts induced by Dex in which ERK ½ pathways have a protective effect, and D1R promotes bone formation that was previously lost due to Dex
Brandon, et al. [72]	Mice	SD rats were injected with a vehicle or MP and tissue was prepared for histochemistry and autoradiogram	The acute and chronic use of MP resulted in the attenuation of osteoclast genes, c-fos, and zif268
Konradi, et al. [79]	Rat	SD rats were subjected to AMP, and brain samples were collected to be tested via immunohistochemistry and electrophoretic mobility-shift assays	AMP induced CREB phosphorylation in the striatum that was blocked via D1R/D5R antagonist
Uddin, et al. [81]	Rat	SD rats were divided into control, low-dose MP, high-dose MP, and pair-fed groups to be tested through an open-field test, caliper measurements, biomechanics, micro-CT, histomorphometry, ex vivo and in vitro TRAP staining, and pit assay	Reduction in weight gain and increases in locomotive activity were noted with MP use, along with negative effects on biomechanics and a male sensitivity of MP on osteoclast differentiation and activity compared to female rats
Chirokikh, et al. [82]	Rat	SD rats were subjected to MP and/or fluoxetine (FLX) and screened for bone morphology and biomechanical properties	MP + FLX rats had shorter and narrower femora and tibia along with a reduction in biomechanical properties and BMC and BMD
Gökçe Nur Say, et al. [52]	Rat	Male Wister–Albino rats were divided into control, low-dose MP, and high-dose MP groups for 13 weeks and then evaluated for weight and biomechanical properties	Dose-dependent effects were noted in weight gain reduction, femur length/size, BMC, and BMD

**Table 5:** Reported effects of growth rate, height, bone mineral density (BMD) and bone mineral content (BMC) within humans reported using psychostimulants.

Reference	Subject	Study Design	Findings
Feuer, et al. [58]	Human	Cross-sectional analysis of data collected from children ages 8–20 with DXA; demographic and prescription medication data available (*n* = 6498)	Patients with reported stimulant use had reduced BMC and BMD of the lumbar spine and femur
Swanson, et al. [59]	Human	Using the Preschool ADHD Treatment Study (PATS), height and weight were recorded 29 times (*n* = 140) over about 1.3 years	Children who continued the use of medication had a decrease in growth rate compared to children not using medication
National Institute [60]	Human	Patients were evaluated longitudinally using a 14- to 24-month assessment of symptoms and growth	The continued use of medication will result in continued maintenance of symptoms and growth suppression
Ortiz, et al. [84]	Human	The optical density technique was used to assess traumatic fractures of the distal radius within a pediatric population at the time of fracture and when the fracture healed (*n* = 188)	Patients using psychostimulant medication for 3–5 years had a negative effect on healing, whereas more than five years of use had no effect on healing
Calarge, et al. [85]	Human	Observational and longitudinal studies of male adolescents taking risperidone (*n* = 194)	There was no significant difference found between boys chronically using MP and those who never used it; however, those using MP had reduced height
Lawson, et al. [86]	Human	Retrospective study of adults using medications for ADHD with DXA scans (*n* = 7961)	BMD was found to be decreased in the skull and thoracic spine of adults using ADHD medication
Diez-Suarez, et al. [88]	Human	An observational longitudinal study of pediatric patients (ages 6–18) diagnosed with ADHD and with MP (*n* = 342)	Decreased weight was noted over 2.2 years of use, whereas height was noted to decrease if MP was started before age 12
Ben-Ami, et al. [87]	Human	Retrospective study of combat soldiers diagnosed with at least one stress fracture diagnosed by a bone scan and with reported exposure to MP (*n* = 100,000)	The majority of soldiers diagnosed with a stress fracture reported the use of MP
